# Tris(1,10-phenanthroline-κ^2^ *N*,*N*′)iron(II) bis­(1,1-dicyano-2-eth­oxy-2-oxoethanide)

**DOI:** 10.1107/S1600536812026967

**Published:** 2012-06-20

**Authors:** Zhan-Mao Cai, Shu-Zhong Zhan

**Affiliations:** a College of Chemistry and Chemical Engineering, South China University of Technology, Guangzhou 510640, People’s Republic of China

## Abstract

The title compound, [Fe(C_12_H_8_N_2_)_3_](C_6_H_5_N_2_O_2_)_2_, consists of one [Fe(phen)_3_]^2+^ cation (phen = 1,10-phenanthroline) and two 1,1-dicyano-2-eth­oxy-2-oxoethanide anions. Five atoms of the anion are disordered over two positions [site occupancy = 0.521 (13) for the major component]. In the complex cation, the Fe^II^ atom is coordinated by six N atoms from three phen ligands in a distorted octa­hedral geometry. Two intra­molecular C—H⋯N hydrogen bonds occur in the complex cation. The crystal structure is mainly stabilized by Coulombic inter­actions. Weak intermolecular C—H⋯N inter­actions are also observed.

## Related literature

For tetra­cyano­ethyl­ene (TCNE) mol­ecular reactions, see: Kaim & Moscherosch (1994 ▶). For geometrical parameters of TCNE, see: Miller (2006 ▶). For the synthesis of the dicyano­ethyl­acetate anion, see: Lv *et al.* (2008 ▶). For the structure of free TCNE, see: Drück & Güth (1982 ▶). For a related structure, see: Uçar *et al.* (2005 ▶).
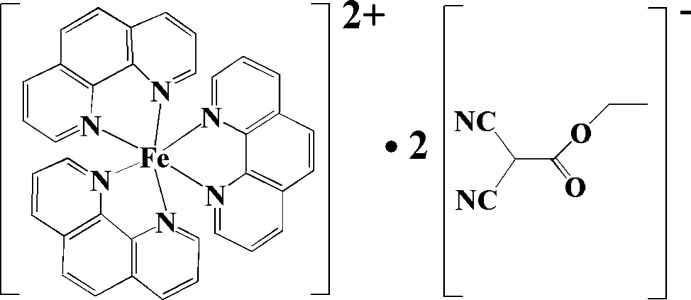


## Experimental

### 

#### Crystal data

[Fe(C_12_H_8_N_2_)_3_](C_6_H_5_N_2_O_2_)_2_
*M* *_r_* = 870.70
Monoclinic, 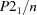

*a* = 15.5855 (5) Å
*b* = 13.0261 (4) Å
*c* = 21.4979 (6) Å
β = 109.068 (1)°
*V* = 4125.0 (2) Å^3^
*Z* = 4
Mo *K*α radiation
μ = 0.43 mm^−1^
*T* = 296 K
0.20 × 0.10 × 0.10 mm

#### Data collection

Bruker APEXII diffractometer
Absorption correction: multi-scan (*SADABS*; Sheldrick, 1996 ▶) *T*_min_ = 0.920, *T*_max_ = 0.959
36630 measured reflections
8551 independent reflections
4862 reflections with *I* > 2σ(*I*)
*R*_int_ = 0.081

#### Refinement

*R*[*F* ^2^ > 2σ(*F* ^2^)] = 0.055
*wR*(*F* ^2^) = 0.156
*S* = 1.02
8551 reflections
614 parameters
71 restraints
H-atom parameters constrained
Δρ_max_ = 0.40 e Å^−3^
Δρ_min_ = −0.44 e Å^−3^

### 

Data collection: *APEX2* (Bruker, 2005 ▶); cell refinement: *SAINT* (Bruker, 2005 ▶); data reduction: *SAINT*; program(s) used to solve structure: *SHELXS97* (Sheldrick, 2008 ▶); program(s) used to refine structure: *SHELXL97* (Sheldrick, 2008 ▶); molecular graphics: *ORTEP-3 for Windows* (Farrugia, 1997 ▶); software used to prepare material for publication: *publCIF* (Westrip, 2010 ▶).

## Supplementary Material

Crystal structure: contains datablock(s) I, global. DOI: 10.1107/S1600536812026967/bx2413sup1.cif

Structure factors: contains datablock(s) I. DOI: 10.1107/S1600536812026967/bx2413Isup2.hkl

Supplementary material file. DOI: 10.1107/S1600536812026967/bx2413Isup3.cdx

Additional supplementary materials:  crystallographic information; 3D view; checkCIF report

## Figures and Tables

**Table 1:** Hydrogen-bond geometry (Å, °)

*D*—H⋯*A*	*D*—H	H⋯*A*	*D*⋯*A*	*D*—H⋯*A*
C25—H25⋯N2	0.93	2.62	3.089 (4)	112
C34—H34⋯N3	0.93	2.60	3.078 (4)	113
C3—H3⋯N8^i^	0.93	2.47	3.206 (6)	136

Symmetry code: (i) 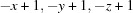.

**Fig. 1.**
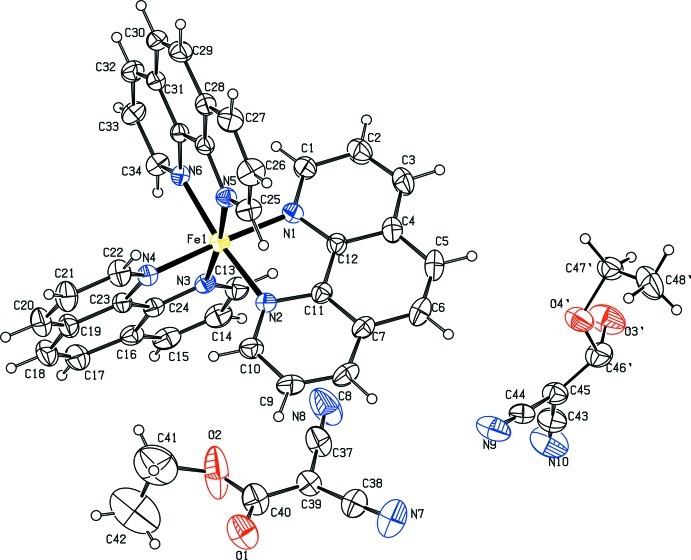
ORTEP view of the title compound, at the 30% probability level.

## supplementary crystallographic information

### Comment

Tetracyanoethylene(TCNE) molecule is one of the most versatile organic compounds as it is used in a many different of reactions (Kaim & Moscherosch, 1994), due to its very low-lying p* orbital. Our interest focus on the reactivity of TCNE and transitionmetal complexes to form discrete as well as polymeric charge-transfer compoundsin which the donors and acceptors are coordinated through nitrile positions. With this mind, we have tried the reaction of FeCl_3_×6H_2_O, 1,10-phenanthroline and TCNE, surprisingly, the title complex {[Fe^II^(phen)_3_][(NC)_2_C—CO_2_C_2_H_5_]} is obtained. In the presence of H_2_O, TCNE can react with ethanol to give dicyanoethylacetate anion-radical (Lv, *et al.*, 2008). The title complex consists of one [Fe^II^(phen)_3_]^2+^ cation, and two dicyanoethylacetate anion-radical. The CN distances are normal range from 1.139 (5) to 1.154 (5) Å. The average C—CN distance of 1.400 (6) Å is 0.035 Å shorter than that observed for the free TCNE (1.435 Å) (Drück & Güth, 1982). The NC—C—CN bond angle are 118.5 (3) and 122.5 (4)*^o^*, which are longer than observed in free TCNE (116.5 (12)*^o^*) in accord with its *sp*^2^ central carbon atom (Miller, 2006; Lv, *et al.*, 2008). In the cation, the Fe^II^ atom is coordinated by six N atoms from three phen ligands in a distorted octahedral geometry.The average bond length of Fe—N is 1.973 (3) Å and similar to tris(1,10-phenanthroline-^2^*N*,*N*')iron(II) squarate octahydrate (Uçar, *et al.*, 2005) as representative example.The crystal structure is mainly stabilized by coulombic interactions. Weak C—H ···N and C—H ···F interactions are also observed, See Table 1.

### Experimental

After addition of tetracyanoethylene (0.261 g, 2 mmol) in ethanol (10 ml) to the solution containing FeCl_3_×6H_2_O(0.270 g, 1 mmol) and 1,10-phenanthroline (phen)(0.400 g, 2 mmol) in ethanol (10 ml), the mixture was stirred at room temperature for 1 h. The solution color turned from red to brown. Single crystals were obtained from the filtrate which was allowed to stand at room temperature for several days, collected by filtration, and dried in *vacuo* (0.26 g, 29.5%). Calcd for C_48_H_34_Fe_2_N_10_O_4_:*C* 66.15, H 3.91, N 16.08. Found: C 65.95, H 3.93, N 16.10.

### Refinement

H atoms were positioned geometrically and refined using a riding model, with C—H = 0.93–0.97 Å and with *U*_iso_(H) = 1.2 (1.5 for methyl groups) times *U*_eq_(C).The C and O atoms of the ethyl formate anion (C46, C47, C48, O3, O4, C46', C47', C48', O3', O4') are disordered over two positions with a refined site-occupancy ratio of 0.521 (13)/0.479 (13). The geometric parameters of two disordered components in each groups were restrained by using SADI restraints and using ISOR constraints. The bond lengths of the disordered atoms were restrained by using *DFIX* instructions. All non-hydrogen atoms were treated anisotropically.

### Crystal data

**Table:**

[Fe(C_12_H_8_N_2_)_3_](C_6_H_5_N_2_O_2_)_2_	*F*(000) = 1800
*M**_r_* = 870.70	*D*_x_ = 1.402 Mg m^−^^3^
Monoclinic, *P*2_1_/*n*	Mo *K*α radiation, λ = 0.71073 Å
*a* = 15.5855 (5) Å	Cell parameters from 36630 reflections
*b* = 13.0261 (4) Å	θ = 1.9–26.5°
*c* = 21.4979 (6) Å	µ = 0.43 mm^−^^1^
β = 109.068 (1)°	*T* = 296 K
*V* = 4125.0 (2) Å^3^	Block, brown
*Z* = 4	0.20 × 0.10 × 0.10 mm

### Data collection

**Table:**

Bruker APEXII diffractometer	4862 reflections with *I* > 2σ(*I*)
Radiation source: fine-focus sealed tube	*R*_int_ = 0.081
Graphite monochromator	θ_max_ = 26.5°, θ_min_ = 1.9°
ω scans	*h* = −17→19
Absorption correction: multi-scan (*SADABS*; Sheldrick, 1996)	*k* = −15→16
*T*_min_ = 0.920, *T*_max_ = 0.959	*l* = −26→26
36630 measured reflections	2 standard reflections every 0 reflections
8551 independent reflections	intensity decay: none

### Refinement

**Table:**

Refinement on *F*^2^	Primary atom site location: structure-invariant direct methods
Least-squares matrix: full	Secondary atom site location: difference Fourier map
*R*[*F*^2^ > 2σ(*F*^2^)] = 0.055	Hydrogen site location: inferred from neighbouring sites
*wR*(*F*^2^) = 0.156	H-atom parameters constrained
*S* = 1.02	*w* = 1/[σ^2^(*F*_o_^2^) + (0.072*P*)^2^] where *P* = (*F*_o_^2^ + 2*F*_c_^2^)/3
8551 reflections	(Δ/σ)_max_ = 0.001
614 parameters	Δρ_max_ = 0.40 e Å^−^^3^
71 restraints	Δρ_min_ = −0.44 e Å^−^^3^

### Special details

**Table:**

Geometry. All e.s.d.'s (except the e.s.d. in the dihedral angle between two l.s. planes) are estimated using the full covariance matrix. The cell e.s.d.'s are taken into account individually in the estimation of e.s.d.'s in distances, angles and torsion angles; correlations between e.s.d.'s in cell parameters are only used when they are defined by crystal symmetry. An approximate (isotropic) treatment of cell e.s.d.'s is used for estimating e.s.d.'s involving l.s. planes.
Refinement. Refinement of *F*^2^ against ALL reflections. The weighted *R*-factor *wR* and goodness of fit *S* are based on *F*^2^, conventional *R*-factors *R* are based on *F*, with *F* set to zero for negative *F*^2^. The threshold expression of *F*^2^ > σ(*F*^2^) is used only for calculating *R*-factors(gt) *etc*. and is not relevant to the choice of reflections for refinement. *R*-factors based on *F*^2^ are statistically about twice as large as those based on *F*, and *R*- factors based on ALL data will be even larger.

### Fractional atomic coordinates and isotropic or equivalent isotropic displacement parameters (Å^2^)

**Table:**

	*x*	*y*	*z*	*U*_iso_*/*U*_eq_	Occ. (<1)
C1	0.6345 (3)	0.3762 (3)	0.44089 (16)	0.0541 (9)	
H1	0.6799	0.3873	0.4224	0.065*	
C2	0.5468 (3)	0.4080 (3)	0.40622 (19)	0.0655 (11)	
H2	0.5348	0.4405	0.3657	0.079*	
C3	0.4785 (3)	0.3919 (3)	0.43135 (19)	0.0651 (11)	
H3	0.4194	0.4111	0.4076	0.078*	
C4	0.4982 (3)	0.3459 (3)	0.49352 (18)	0.0544 (9)	
C5	0.4325 (3)	0.3255 (3)	0.5255 (2)	0.0691 (11)	
H5	0.3719	0.3421	0.5044	0.083*	
C6	0.4576 (3)	0.2826 (3)	0.5858 (2)	0.0656 (11)	
H6	0.4138	0.2710	0.6057	0.079*	
C7	0.5496 (3)	0.2542 (3)	0.62009 (17)	0.0508 (9)	
C8	0.5801 (3)	0.2082 (3)	0.6821 (2)	0.0650 (11)	
H8	0.5395	0.1943	0.7046	0.078*	
C9	0.6692 (3)	0.1840 (3)	0.70946 (18)	0.0635 (11)	
H9	0.6899	0.1540	0.7510	0.076*	
C10	0.7298 (3)	0.2040 (3)	0.67522 (16)	0.0521 (9)	
H10	0.7907	0.1871	0.6949	0.063*	
C11	0.6143 (2)	0.2716 (2)	0.58900 (15)	0.0425 (8)	
C12	0.5876 (2)	0.3171 (2)	0.52548 (15)	0.0429 (8)	
C13	0.7824 (3)	0.4959 (3)	0.58974 (17)	0.0511 (9)	
H13	0.7223	0.5022	0.5629	0.061*	
C14	0.8288 (3)	0.5838 (3)	0.61999 (18)	0.0593 (10)	
H14	0.7997	0.6472	0.6130	0.071*	
C15	0.9162 (3)	0.5766 (3)	0.65943 (18)	0.0564 (10)	
H15	0.9473	0.6353	0.6791	0.068*	
C16	0.9600 (2)	0.4812 (3)	0.67077 (16)	0.0473 (9)	
C17	1.0508 (3)	0.4632 (3)	0.71111 (18)	0.0644 (11)	
H17	1.0865	0.5184	0.7320	0.077*	
C18	1.0870 (3)	0.3674 (3)	0.72003 (18)	0.0644 (11)	
H18	1.1469	0.3585	0.7469	0.077*	
C19	1.0352 (2)	0.2796 (3)	0.68906 (17)	0.0517 (9)	
C20	1.0665 (3)	0.1785 (3)	0.69839 (19)	0.0648 (11)	
H20	1.1253	0.1641	0.7255	0.078*	
C21	1.0102 (3)	0.1015 (3)	0.6674 (2)	0.0645 (11)	
H21	1.0298	0.0338	0.6739	0.077*	
C22	0.9227 (3)	0.1240 (3)	0.62577 (18)	0.0539 (9)	
H22	0.8857	0.0700	0.6045	0.065*	
C23	0.9460 (2)	0.2961 (2)	0.64793 (15)	0.0405 (8)	
C24	0.9083 (2)	0.3960 (2)	0.63876 (15)	0.0396 (8)	
C25	0.6919 (2)	0.0594 (3)	0.52483 (17)	0.0526 (9)	
H25	0.6742	0.0639	0.5621	0.063*	
C26	0.6700 (3)	−0.0289 (3)	0.4865 (2)	0.0639 (11)	
H26	0.6390	−0.0822	0.4986	0.077*	
C27	0.6940 (3)	−0.0369 (3)	0.43108 (18)	0.0591 (10)	
H27	0.6781	−0.0950	0.4046	0.071*	
C28	0.7426 (2)	0.0423 (2)	0.41425 (15)	0.0452 (8)	
C29	0.7748 (3)	0.0411 (3)	0.35912 (16)	0.0541 (9)	
H29	0.7628	−0.0156	0.3313	0.065*	
C30	0.8218 (2)	0.1198 (3)	0.34666 (16)	0.0519 (9)	
H30	0.8417	0.1163	0.3104	0.062*	
C31	0.8423 (2)	0.2094 (2)	0.38756 (14)	0.0420 (8)	
C32	0.8902 (2)	0.2946 (3)	0.37698 (16)	0.0529 (9)	
H32	0.9109	0.2965	0.3411	0.063*	
C33	0.9061 (3)	0.3750 (3)	0.41977 (17)	0.0536 (9)	
H33	0.9377	0.4322	0.4131	0.064*	
C34	0.8749 (2)	0.3714 (2)	0.47350 (16)	0.0471 (8)	
H34	0.8868	0.4272	0.5021	0.056*	
C35	0.8125 (2)	0.2118 (2)	0.44221 (14)	0.0372 (7)	
C36	0.7623 (2)	0.1283 (2)	0.45555 (14)	0.0385 (7)	
C37	0.6703 (3)	0.4689 (4)	0.7487 (2)	0.0763 (12)	
C38	0.6090 (3)	0.3700 (3)	0.8186 (2)	0.0660 (11)	
C39	0.6846 (3)	0.4054 (3)	0.80393 (18)	0.0583 (10)	
C40	0.7696 (3)	0.3642 (3)	0.8385 (2)	0.0757 (13)	
C41	0.9236 (5)	0.3382 (6)	0.8253 (4)	0.165 (3)	
H41A	0.9184	0.2670	0.8368	0.197*	
H41B	0.9462	0.3427	0.7883	0.197*	
C42	0.9732 (6)	0.3985 (6)	0.8793 (4)	0.200 (4)	
H42A	1.0345	0.3738	0.8960	0.300*	
H42B	0.9457	0.3942	0.9132	0.300*	
H42C	0.9732	0.4686	0.8655	0.300*	
C43	0.2814 (3)	0.4440 (3)	0.66824 (19)	0.0667 (11)	
C44	0.3051 (3)	0.2615 (3)	0.68039 (17)	0.0541 (9)	
C45	0.2585 (3)	0.3446 (3)	0.64328 (17)	0.0560 (10)	
Fe1	0.77305 (3)	0.27189 (3)	0.55530 (2)	0.03744 (16)	
N1	0.65628 (18)	0.33070 (19)	0.49930 (12)	0.0418 (6)	
N2	0.70377 (19)	0.24633 (19)	0.61563 (12)	0.0413 (7)	
N3	0.82099 (19)	0.40325 (18)	0.59777 (12)	0.0393 (6)	
N4	0.88960 (18)	0.21919 (18)	0.61503 (12)	0.0397 (6)	
N5	0.73710 (18)	0.13796 (19)	0.51064 (12)	0.0409 (6)	
N6	0.82885 (17)	0.29195 (18)	0.48604 (12)	0.0372 (6)	
N7	0.5459 (3)	0.3409 (3)	0.8287 (2)	0.0994 (14)	
N8	0.6593 (3)	0.5196 (4)	0.7032 (2)	0.1231 (18)	
N9	0.3453 (3)	0.1940 (3)	0.70941 (16)	0.0832 (12)	
N10	0.3005 (3)	0.5257 (3)	0.68888 (19)	0.1009 (14)	
O1	0.7877 (2)	0.3023 (2)	0.88383 (16)	0.0938 (10)	
O2	0.8340 (3)	0.4014 (4)	0.8148 (2)	0.157 (2)	
C46	0.2022 (7)	0.3307 (8)	0.5764 (4)	0.053 (4)	0.479 (13)
C47	0.0833 (11)	0.2138 (9)	0.5114 (7)	0.105 (5)	0.479 (13)
H47A	0.0920	0.2512	0.4749	0.126*	0.479 (13)
H47B	0.0288	0.2400	0.5183	0.126*	0.479 (13)
C48	0.0702 (12)	0.1014 (9)	0.4937 (8)	0.097 (5)	0.479 (13)
H48A	0.0112	0.0911	0.4619	0.146*	0.479 (13)
H48B	0.0754	0.0620	0.5326	0.146*	0.479 (13)
H48C	0.1159	0.0795	0.4756	0.146*	0.479 (13)
O3	0.1259 (6)	0.3932 (8)	0.5550 (5)	0.079 (3)	0.479 (13)
O4	0.1607 (7)	0.2316 (9)	0.5704 (5)	0.061 (3)	0.479 (13)
C46'	0.1785 (7)	0.3322 (10)	0.5883 (4)	0.069 (4)	0.521 (13)
C47'	0.1297 (7)	0.2109 (7)	0.4935 (4)	0.064 (3)	0.521 (13)
H47C	0.1612	0.1765	0.4673	0.076*	0.521 (13)
H47D	0.1020	0.2731	0.4710	0.076*	0.521 (13)
C48'	0.0582 (9)	0.1405 (12)	0.5053 (8)	0.095 (4)	0.521 (13)
H48D	0.0184	0.1158	0.4639	0.142*	0.521 (13)
H48E	0.0237	0.1781	0.5274	0.142*	0.521 (13)
H48F	0.0876	0.0834	0.5320	0.142*	0.521 (13)
O3'	0.1696 (10)	0.4051 (7)	0.5404 (4)	0.099 (3)	0.521 (13)
O4'	0.1919 (8)	0.2343 (9)	0.5587 (5)	0.074 (3)	0.521 (13)

### Atomic displacement parameters (Å^2^)

**Table:**

	*U*^11^	*U*^22^	*U*^33^	*U*^12^	*U*^13^	*U*^23^
C1	0.053 (3)	0.060 (2)	0.048 (2)	0.0086 (19)	0.0151 (18)	0.0114 (17)
C2	0.063 (3)	0.070 (3)	0.057 (2)	0.010 (2)	0.011 (2)	0.0147 (19)
C3	0.048 (3)	0.069 (3)	0.068 (3)	0.015 (2)	0.004 (2)	0.008 (2)
C4	0.041 (2)	0.054 (2)	0.065 (2)	0.0016 (18)	0.0134 (19)	−0.0042 (17)
C5	0.038 (3)	0.074 (3)	0.094 (3)	0.006 (2)	0.020 (2)	0.000 (2)
C6	0.053 (3)	0.068 (3)	0.087 (3)	−0.001 (2)	0.039 (2)	−0.001 (2)
C7	0.052 (3)	0.052 (2)	0.056 (2)	−0.0057 (18)	0.0281 (19)	−0.0062 (16)
C8	0.072 (3)	0.073 (3)	0.067 (3)	−0.007 (2)	0.046 (2)	−0.002 (2)
C9	0.079 (3)	0.073 (3)	0.042 (2)	−0.002 (2)	0.027 (2)	0.0065 (18)
C10	0.058 (3)	0.057 (2)	0.0433 (19)	−0.0047 (18)	0.0190 (18)	0.0011 (15)
C11	0.044 (2)	0.0387 (18)	0.0463 (19)	−0.0010 (16)	0.0173 (16)	−0.0053 (14)
C12	0.039 (2)	0.0422 (18)	0.0475 (19)	−0.0017 (16)	0.0137 (16)	−0.0046 (14)
C13	0.058 (3)	0.042 (2)	0.055 (2)	0.0050 (18)	0.0198 (18)	−0.0033 (16)
C14	0.079 (3)	0.042 (2)	0.064 (2)	0.004 (2)	0.033 (2)	−0.0053 (17)
C15	0.078 (3)	0.041 (2)	0.060 (2)	−0.015 (2)	0.036 (2)	−0.0143 (16)
C16	0.053 (3)	0.050 (2)	0.0442 (19)	−0.0133 (18)	0.0227 (18)	−0.0078 (15)
C17	0.054 (3)	0.070 (3)	0.065 (3)	−0.023 (2)	0.014 (2)	−0.014 (2)
C18	0.043 (3)	0.078 (3)	0.063 (2)	−0.011 (2)	0.0060 (19)	−0.004 (2)
C19	0.039 (2)	0.060 (2)	0.051 (2)	−0.0027 (19)	0.0091 (17)	0.0015 (17)
C20	0.044 (3)	0.067 (3)	0.074 (3)	0.007 (2)	0.008 (2)	0.013 (2)
C21	0.054 (3)	0.049 (2)	0.086 (3)	0.014 (2)	0.016 (2)	0.013 (2)
C22	0.055 (3)	0.0355 (19)	0.068 (2)	0.0039 (17)	0.016 (2)	0.0051 (16)
C23	0.036 (2)	0.047 (2)	0.0389 (17)	−0.0058 (15)	0.0121 (15)	−0.0028 (14)
C24	0.044 (2)	0.0392 (18)	0.0383 (17)	−0.0023 (16)	0.0171 (16)	−0.0032 (13)
C25	0.064 (3)	0.044 (2)	0.053 (2)	−0.0112 (18)	0.0235 (19)	−0.0044 (16)
C26	0.076 (3)	0.042 (2)	0.078 (3)	−0.017 (2)	0.030 (2)	−0.0052 (18)
C27	0.065 (3)	0.044 (2)	0.065 (2)	−0.0106 (19)	0.018 (2)	−0.0161 (17)
C28	0.043 (2)	0.0416 (19)	0.048 (2)	0.0016 (16)	0.0105 (16)	−0.0062 (15)
C29	0.058 (3)	0.054 (2)	0.046 (2)	0.0073 (19)	0.0103 (18)	−0.0144 (16)
C30	0.049 (2)	0.065 (2)	0.0411 (19)	0.0140 (19)	0.0142 (17)	−0.0026 (16)
C31	0.038 (2)	0.049 (2)	0.0363 (17)	0.0077 (16)	0.0093 (15)	0.0003 (14)
C32	0.054 (3)	0.064 (2)	0.047 (2)	0.0060 (19)	0.0236 (18)	0.0102 (17)
C33	0.060 (3)	0.052 (2)	0.058 (2)	−0.0038 (19)	0.0319 (19)	0.0051 (17)
C34	0.054 (2)	0.0381 (18)	0.052 (2)	−0.0023 (17)	0.0224 (18)	0.0015 (15)
C35	0.036 (2)	0.0394 (18)	0.0348 (16)	0.0074 (14)	0.0092 (14)	0.0008 (13)
C36	0.037 (2)	0.0387 (17)	0.0372 (17)	0.0044 (15)	0.0082 (14)	−0.0001 (13)
C37	0.060 (3)	0.078 (3)	0.080 (3)	−0.007 (2)	0.008 (2)	0.013 (2)
C38	0.071 (3)	0.047 (2)	0.084 (3)	0.002 (2)	0.031 (3)	−0.0013 (19)
C39	0.059 (3)	0.051 (2)	0.061 (2)	−0.004 (2)	0.015 (2)	0.0043 (18)
C40	0.066 (3)	0.065 (3)	0.091 (3)	−0.010 (2)	0.019 (3)	0.018 (2)
C41	0.185 (9)	0.158 (7)	0.139 (6)	−0.006 (7)	0.037 (6)	0.052 (6)
C42	0.229 (10)	0.144 (7)	0.173 (8)	−0.026 (7)	−0.007 (7)	0.016 (6)
C43	0.083 (3)	0.058 (3)	0.057 (2)	−0.003 (2)	0.019 (2)	0.0041 (19)
C44	0.065 (3)	0.056 (2)	0.0398 (19)	−0.005 (2)	0.0150 (18)	−0.0088 (18)
C45	0.067 (3)	0.049 (2)	0.050 (2)	0.0041 (19)	0.016 (2)	−0.0046 (17)
Fe1	0.0397 (3)	0.0350 (3)	0.0381 (3)	−0.0002 (2)	0.0134 (2)	−0.00014 (19)
N1	0.0431 (18)	0.0413 (15)	0.0392 (14)	−0.0023 (13)	0.0112 (13)	−0.0004 (11)
N2	0.0422 (19)	0.0451 (16)	0.0369 (14)	−0.0004 (13)	0.0132 (13)	−0.0015 (11)
N3	0.0465 (18)	0.0328 (14)	0.0419 (15)	0.0044 (12)	0.0191 (13)	−0.0003 (11)
N4	0.0405 (17)	0.0355 (15)	0.0430 (15)	−0.0027 (13)	0.0136 (12)	0.0007 (11)
N5	0.0419 (18)	0.0404 (15)	0.0396 (14)	−0.0020 (13)	0.0123 (13)	−0.0026 (11)
N6	0.0349 (16)	0.0364 (15)	0.0408 (14)	0.0012 (12)	0.0129 (12)	0.0001 (11)
N7	0.096 (4)	0.070 (3)	0.151 (4)	0.008 (2)	0.065 (3)	0.017 (2)
N8	0.092 (3)	0.151 (4)	0.103 (3)	−0.014 (3)	0.000 (3)	0.065 (3)
N9	0.117 (3)	0.060 (2)	0.054 (2)	0.011 (2)	0.003 (2)	−0.0038 (17)
N10	0.135 (4)	0.059 (2)	0.089 (3)	−0.020 (2)	0.011 (3)	−0.007 (2)
O1	0.090 (2)	0.078 (2)	0.099 (2)	−0.0072 (17)	0.0109 (19)	0.0327 (18)
O2	0.060 (3)	0.169 (4)	0.237 (5)	0.023 (3)	0.039 (3)	0.130 (4)
C46	0.070 (7)	0.052 (6)	0.042 (5)	0.027 (5)	0.025 (5)	0.003 (4)
C47	0.101 (9)	0.081 (7)	0.103 (8)	0.014 (7)	−0.008 (6)	−0.028 (6)
C48	0.092 (9)	0.089 (8)	0.095 (8)	0.008 (7)	0.010 (6)	−0.017 (6)
O3	0.082 (6)	0.057 (4)	0.079 (5)	0.017 (4)	−0.003 (4)	0.000 (4)
O4	0.052 (5)	0.058 (4)	0.059 (5)	0.014 (4)	−0.002 (3)	−0.020 (3)
C46'	0.083 (8)	0.060 (6)	0.066 (6)	0.023 (5)	0.026 (6)	−0.010 (5)
C47'	0.070 (6)	0.066 (5)	0.049 (4)	0.015 (4)	0.013 (4)	0.002 (3)
C48'	0.074 (7)	0.087 (8)	0.096 (8)	0.021 (7)	−0.009 (5)	0.001 (7)
O3'	0.136 (8)	0.066 (4)	0.074 (4)	0.019 (5)	0.006 (5)	0.012 (3)
O4'	0.087 (7)	0.064 (4)	0.055 (4)	0.026 (5)	0.004 (4)	−0.010 (3)

### Geometric parameters (Å, º)

**Table:**

C1—N1	1.329 (4)	C29—H29	0.9300
C1—C2	1.389 (5)	C30—C31	1.433 (5)
C1—H1	0.9300	C30—H30	0.9300
C2—C3	1.357 (5)	C31—C32	1.396 (5)
C2—H2	0.9300	C31—C35	1.398 (4)
C3—C4	1.404 (5)	C32—C33	1.362 (5)
C3—H3	0.9300	C32—H32	0.9300
C4—C12	1.389 (5)	C33—C34	1.392 (4)
C4—C5	1.432 (5)	C33—H33	0.9300
C5—C6	1.347 (5)	C34—N6	1.337 (4)
C5—H5	0.9300	C34—H34	0.9300
C6—C7	1.429 (5)	C35—N6	1.373 (4)
C6—H6	0.9300	C35—C36	1.423 (4)
C7—C8	1.396 (5)	C36—N5	1.370 (4)
C7—C11	1.399 (4)	C37—N8	1.147 (5)
C8—C9	1.356 (5)	C37—C39	1.403 (6)
C8—H8	0.9300	C38—N7	1.139 (5)
C9—C10	1.400 (5)	C38—C39	1.394 (6)
C9—H9	0.9300	C39—C40	1.398 (6)
C10—N2	1.330 (4)	C40—O1	1.224 (5)
C10—H10	0.9300	C40—O2	1.356 (5)
C11—N2	1.363 (4)	C41—C42	1.405 (7)
C11—C12	1.421 (4)	C41—O2	1.573 (7)
C12—N1	1.374 (4)	C41—H41A	0.9700
C13—N3	1.334 (4)	C41—H41B	0.9700
C13—C14	1.397 (5)	C42—H42A	0.9600
C13—H13	0.9300	C42—H42B	0.9600
C14—C15	1.352 (5)	C42—H42C	0.9600
C14—H14	0.9300	C43—N10	1.154 (5)
C15—C16	1.401 (5)	C43—C45	1.403 (5)
C15—H15	0.9300	C44—N9	1.139 (5)
C16—C24	1.411 (4)	C44—C45	1.399 (5)
C16—C17	1.417 (5)	C45—C46'	1.420 (8)
C17—C18	1.357 (5)	C45—C46	1.431 (8)
C17—H17	0.9300	Fe1—N2	1.968 (2)
C18—C19	1.432 (5)	Fe1—N3	1.968 (3)
C18—H18	0.9300	Fe1—N6	1.972 (2)
C19—C20	1.396 (5)	Fe1—N4	1.974 (3)
C19—C23	1.399 (5)	Fe1—N1	1.979 (3)
C20—C21	1.355 (5)	Fe1—N5	1.982 (2)
C20—H20	0.9300	C46—O3	1.390 (9)
C21—C22	1.396 (5)	C46—O4	1.431 (16)
C21—H21	0.9300	C47—O4	1.456 (9)
C22—N4	1.334 (4)	C47—C48	1.509 (9)
C22—H22	0.9300	C47—H47A	0.9700
C23—N4	1.368 (4)	C47—H47B	0.9700
C23—C24	1.414 (4)	C48—H48A	0.9600
C24—N3	1.362 (4)	C48—H48B	0.9600
C25—N5	1.332 (4)	C48—H48C	0.9600
C25—C26	1.391 (5)	C46'—O3'	1.375 (9)
C25—H25	0.9300	C46'—O4'	1.470 (17)
C26—C27	1.365 (5)	C47'—O4'	1.452 (8)
C26—H26	0.9300	C47'—C48'	1.526 (9)
C27—C28	1.396 (5)	C47'—H47C	0.9700
C27—H27	0.9300	C47'—H47D	0.9700
C28—C36	1.400 (4)	C48'—H48D	0.9600
C28—C29	1.430 (4)	C48'—H48E	0.9600
C29—C30	1.337 (5)	C48'—H48F	0.9600
			
N1—C1—C2	122.9 (3)	C33—C34—H34	118.4
N1—C1—H1	118.5	N6—C35—C31	123.8 (3)
C2—C1—H1	118.5	N6—C35—C36	115.7 (3)
C3—C2—C1	120.1 (4)	C31—C35—C36	120.5 (3)
C3—C2—H2	119.9	N5—C36—C28	123.8 (3)
C1—C2—H2	119.9	N5—C36—C35	115.9 (3)
C2—C3—C4	119.3 (4)	C28—C36—C35	120.3 (3)
C2—C3—H3	120.3	N8—C37—C39	178.8 (6)
C4—C3—H3	120.3	N7—C38—C39	178.0 (5)
C12—C4—C3	117.2 (3)	C38—C39—C40	118.5 (4)
C12—C4—C5	118.1 (3)	C38—C39—C37	118.3 (4)
C3—C4—C5	124.7 (4)	C40—C39—C37	122.5 (4)
C6—C5—C4	120.7 (4)	O1—C40—O2	121.9 (4)
C6—C5—H5	119.6	O1—C40—C39	127.4 (4)
C4—C5—H5	119.6	O2—C40—C39	110.7 (4)
C5—C6—C7	122.1 (4)	C42—C41—O2	92.8 (7)
C5—C6—H6	119.0	C42—C41—H41A	113.1
C7—C6—H6	119.0	O2—C41—H41A	113.1
C8—C7—C11	116.9 (4)	C42—C41—H41B	113.1
C8—C7—C6	125.1 (3)	O2—C41—H41B	113.1
C11—C7—C6	118.0 (3)	H41A—C41—H41B	110.5
C9—C8—C7	119.7 (3)	C41—C42—H42A	109.5
C9—C8—H8	120.2	C41—C42—H42B	109.5
C7—C8—H8	120.2	H42A—C42—H42B	109.5
C8—C9—C10	120.0 (3)	C41—C42—H42C	109.5
C8—C9—H9	120.0	H42A—C42—H42C	109.5
C10—C9—H9	120.0	H42B—C42—H42C	109.5
N2—C10—C9	122.5 (4)	N10—C43—C45	179.7 (6)
N2—C10—H10	118.7	N9—C44—C45	177.5 (4)
C9—C10—H10	118.7	C44—C45—C43	118.5 (3)
N2—C11—C7	124.0 (3)	C44—C45—C46'	122.5 (6)
N2—C11—C12	116.3 (3)	C43—C45—C46'	117.9 (6)
C7—C11—C12	119.7 (3)	C44—C45—C46	120.6 (5)
N1—C12—C4	123.5 (3)	C43—C45—C46	119.8 (5)
N1—C12—C11	115.1 (3)	C46'—C45—C46	20.8 (8)
C4—C12—C11	121.3 (3)	N2—Fe1—N3	92.77 (10)
N3—C13—C14	122.6 (4)	N2—Fe1—N6	172.88 (11)
N3—C13—H13	118.7	N3—Fe1—N6	92.34 (10)
C14—C13—H13	118.7	N2—Fe1—N4	95.68 (10)
C15—C14—C13	119.8 (4)	N3—Fe1—N4	82.61 (11)
C15—C14—H14	120.1	N6—Fe1—N4	89.89 (10)
C13—C14—H14	120.1	N2—Fe1—N1	82.70 (11)
C14—C15—C16	120.3 (3)	N3—Fe1—N1	94.58 (11)
C14—C15—H15	119.8	N6—Fe1—N1	91.95 (10)
C16—C15—H15	119.8	N4—Fe1—N1	176.71 (10)
C15—C16—C24	116.4 (3)	N2—Fe1—N5	92.36 (10)
C15—C16—C17	125.9 (3)	N3—Fe1—N5	173.92 (11)
C24—C16—C17	117.7 (3)	N6—Fe1—N5	82.84 (10)
C18—C17—C16	121.7 (4)	N4—Fe1—N5	93.64 (10)
C18—C17—H17	119.2	N1—Fe1—N5	89.30 (11)
C16—C17—H17	119.2	C1—N1—C12	116.8 (3)
C17—C18—C19	121.5 (4)	C1—N1—Fe1	130.4 (2)
C17—C18—H18	119.2	C12—N1—Fe1	112.7 (2)
C19—C18—H18	119.2	C10—N2—C11	116.9 (3)
C20—C19—C23	117.7 (3)	C10—N2—Fe1	130.1 (2)
C20—C19—C18	124.7 (4)	C11—N2—Fe1	112.9 (2)
C23—C19—C18	117.6 (3)	C13—N3—C24	117.4 (3)
C21—C20—C19	119.1 (4)	C13—N3—Fe1	129.8 (2)
C21—C20—H20	120.4	C24—N3—Fe1	112.79 (19)
C19—C20—H20	120.4	C22—N4—C23	116.4 (3)
C20—C21—C22	120.0 (4)	C22—N4—Fe1	131.1 (2)
C20—C21—H21	120.0	C23—N4—Fe1	112.4 (2)
C22—C21—H21	120.0	C25—N5—C36	116.7 (3)
N4—C22—C21	123.2 (3)	C25—N5—Fe1	130.7 (2)
N4—C22—H22	118.4	C36—N5—Fe1	112.6 (2)
C21—C22—H22	118.4	C34—N6—C35	116.2 (3)
N4—C23—C19	123.5 (3)	C34—N6—Fe1	130.8 (2)
N4—C23—C24	115.7 (3)	C35—N6—Fe1	112.9 (2)
C19—C23—C24	120.8 (3)	C40—O2—C41	119.4 (4)
N3—C24—C16	123.5 (3)	O3—C46—O4	100.7 (11)
N3—C24—C23	115.8 (3)	O3—C46—C45	115.9 (8)
C16—C24—C23	120.7 (3)	O4—C46—C45	108.4 (8)
N5—C25—C26	123.0 (3)	O4—C47—C48	112.2 (11)
N5—C25—H25	118.5	O4—C47—H47A	109.2
C26—C25—H25	118.5	C48—C47—H47A	109.2
C27—C26—C25	119.7 (3)	O4—C47—H47B	109.2
C27—C26—H26	120.1	C48—C47—H47B	109.2
C25—C26—H26	120.1	H47A—C47—H47B	107.9
C26—C27—C28	119.8 (3)	C47—C48—H48A	109.5
C26—C27—H27	120.1	C47—C48—H48B	109.5
C28—C27—H27	120.1	H48A—C48—H48B	109.5
C27—C28—C36	116.9 (3)	C47—C48—H48C	109.5
C27—C28—C29	125.0 (3)	H48A—C48—H48C	109.5
C36—C28—C29	118.1 (3)	H48B—C48—H48C	109.5
C30—C29—C28	121.4 (3)	C46—O4—C47	116.4 (10)
C30—C29—H29	119.3	O3'—C46'—C45	113.0 (9)
C28—C29—H29	119.3	O3'—C46'—O4'	105.5 (12)
C29—C30—C31	121.9 (3)	C45—C46'—O4'	104.4 (9)
C29—C30—H30	119.0	O4'—C47'—C48'	105.0 (9)
C31—C30—H30	119.0	O4'—C47'—H47C	110.7
C32—C31—C35	117.4 (3)	C48'—C47'—H47C	110.7
C32—C31—C30	124.9 (3)	O4'—C47'—H47D	110.7
C35—C31—C30	117.7 (3)	C48'—C47'—H47D	110.7
C33—C32—C31	119.4 (3)	H47C—C47'—H47D	108.8
C33—C32—H32	120.3	C47'—C48'—H48D	109.5
C31—C32—H32	120.3	C47'—C48'—H48E	109.5
C32—C33—C34	119.9 (3)	H48D—C48'—H48E	109.5
C32—C33—H33	120.0	C47'—C48'—H48F	109.5
C34—C33—H33	120.0	H48D—C48'—H48F	109.5
N6—C34—C33	123.3 (3)	H48E—C48'—H48F	109.5
N6—C34—H34	118.4	C47'—O4'—C46'	117.1 (8)

### Hydrogen-bond geometry (Å, º)

**Table:**

*D*—H···*A*	*D*—H	H···*A*	*D*···*A*	*D*—H···*A*
C25—H25···N2	0.93	2.62	3.089 (4)	112
C34—H34···N3	0.93	2.60	3.078 (4)	113
C3—H3···N8^i^	0.93	2.47	3.206 (6)	136

Symmetry code: (i) −*x*+1, −*y*+1, −*z*+1.
